# Packed Lunch Provision and Consumption in Early Years Settings in Sheffield: A Cross‐Sectional Study

**DOI:** 10.1111/jhn.70066

**Published:** 2025-05-14

**Authors:** Jo Pearce, Claire J. Wall

**Affiliations:** ^1^ Sheffield Business School Sheffield Hallam University Sheffield UK

**Keywords:** children, early years, nursery, nutrient analysis, packed lunch

## Abstract

**Introduction:**

In 2024, 95% of 3–4‐year‐old children in England attended early years settings (EYS). This study aimed to estimate energy and nutrient content of packed lunches provided for and consumed by children attending EYS, along with food type, cost and level of processing and whether these differed by area deprivation.

**Methods:**

An observational cross‐sectional weighed intake and nutrient analysis of food and drinks in packed lunches provided to and consumed by children attending eight EYS over 5 days. Food type, packaging and processing were coded and lunch costs calculated.

**Results:**

A total of 67 children ate 185 packed lunches. Lunches included fruit (76.2%) more often than vegetables (38.9%). Lunches in areas of higher deprivation less frequently contained fruit (*p* = 0.003) or vegetables (*p* < 0.001), and more frequently contained meat products (*p* < 0.001), savoury snacks (*p* < 0.001), cakes/biscuits (*p* = 0.038) and confectionery (*p* < 0.001). Use of pre‐packaged foods was common (40.1% items), and ultra‐processed foods provided 65.5% energy. Overall, lunches provided sufficient fibre and micronutrients, but high protein and excess energy, fat, saturated fat, free sugars and sodium. Provision of most nutrients varied by area deprivation, but consumption in areas of higher deprivation was only higher for free sugars (*p* = 0.002) and lower for fibre (*p* = 0.003) and vitamin C (*p* = 0.003). Median packed lunch cost was £1.26, with no difference by area deprivation (*p* = 0.422). Healthier lunches were cheaper than less healthy lunches (*p* < 0.001).

**Conclusions:**

Use of pre‐packed, ultra‐processed foods was high, and provision of vegetables low. Lunches were high in sodium and free sugars, with less healthy lunches provided in areas of higher deprivation.

## Summary

- The median cost of a packed lunch, for children attending early years settings was £1.26 (mean £1.42) in July 2024. Packed lunches with a higher healthy eating score (£1.23) were significantly cheaper than lunches with a lower healthy eating score (£1.72).
- Packed lunches in more deprived areas contained fewer fruit and vegetables but more meat products, savoury snacks, cakes and biscuits, chocolate or sugar confectionary and squash/fruit drinks.
- Purchased individually pre‐packaged items were provided more frequently than home‐made foods.
- Ultra‐processed foods (NOVA 4 group) constituted 65.5% of the energy provided to children and 67.1% of the energy consumed by children, from lunchboxes.
- Children were provided with food that met lunchtime nutrient recommendations for fibre, vitamin A, vitamin C, calcium, iodine, iron and zinc, regardless of area deprivation.
- Provision of energy, free sugars, sodium, protein, carbohydrate, fat and saturated fat were above recommended levels.
- Children left around 20% of the food provided by their packed lunch.

## 1 Introduction

Adequate nutrition is essential for the health and wellbeing of pre‐school children [1, 2, 3]. In the UK however, 18.7% of households with children reported experiencing food insecurity, with 11.8% relying on low‐cost food, and 9.2% not eating balanced meals [4]. Families are more likely to experience food insecurity when they have a lower income and live in areas of higher deprivation [5].

In 2024, 95% of 3‐ and 4‐year‐olds in England were registered to receive 15 h per week of government funded early education, which includes early years places provided by nurseries, school‐based nursery classes and childminders [6]. With such a high proportion of children attending early years settings (EYS), food provision in settings could help to achieve optimal nutrition and reduce dietary inequalities by ensuring healthy and balanced meals are provided [7]. EYS can also support children to develop life‐long healthy eating habits, food skills and enjoyment of food [8, 9]. EYS often differ from schools in pattern of meal provision in addition to variation in setting type and age of attending children. They may provide all or some of children's meals (breakfast, lunch or tea (evening meal)) and snacks or no food at all, depending on their funding, policies, staff and facilities [10]. If children attend an EYS over lunchtime they could, therefore, consume either a setting‐provided lunch or lunch provided from home (a packed lunch) [11]. Whilst numerous studies in schools have demonstrated packed lunches have a less favourable nutrient profile than school lunches, including for children from low‐income households [12, 13, 14, 15, 16], to date, only one UK study has explored packed lunches provided to children in EYS [11]. When compared to setting‐provided lunches, packed lunches contained more bread‐based items, cakes and biscuits, fruit, squash (cordial), crisps, confectionery, and were higher in energy, non‐milk extrinsic sugars (NMES), sodium, calcium, and vitamin C, and lower in fibre, vitamin A and folate [11]. Similarly, although there is concern about the proportion of ultra‐processed foods (UPF) within the diets of young children [17], no UK studies have explored UPF provision or consumption within EYS. Secondary analysis of national diet and nutrition survey (NDNS) data from primary and secondary school‐aged children found higher levels of UPF (78% and 76% energy respectively) in packed lunches, compared to school lunches [18].

Packed lunches provided to school children are generally perceived to be cheaper than school lunches [19], which are usually tethered to the cost of a free school meal (£2.53 at the time of writing) [20]. A UK charity estimated that a typical ‘healthy’ packed lunch may be an average of 45% more expensive than an ‘unhealthy’ packed lunch, although an Australian study of 1026 lunches found less healthy lunches were more expensive, as were lunches provided to children in the most disadvantaged households, possibly due to the higher prevalence of more costly pre‐packed items [21, 22].

Given the lack of research on packed lunches in EYS, the aim of this study was to estimate the energy and nutrient content of a sample of packed lunches provided for and eaten by children attending EYS, the cost of the packed lunches provided, the types of food provided, including the level of processing, and whether these varied by area deprivation.

## 2 Materials and Methods

### 2.1 EYS Recruitment

Ethical approval was granted by Sheffield Hallam University research ethics review system (ID:ER61543155). EYS within Sheffield were identified in February 2024. EYS listed as providing sessional or full day childcare on non‐domestic premises (e.g., private and community nurseries, pre‐schools), but excluding out of school care (e.g., holiday clubs) were identified from the Ofsted early years register [23]. Primary and infant schools with nursery classes were identified from the government school database [24]. In total, 123 EYS and 79 schools were identified, contacted by email and invited to participate. Two schools with nursery/pre‐school classes initially consented to take part. Purposive sampling was then used to contact and recruit a further six EYS to represent variety in EYS type and area deprivation.

Participating EYS sent information and paper and/or online consent forms to parents/caregivers of all 3–4‐year‐old children who stayed for lunch at least 1 day a week. Alongside consent, each child's age and sex were also collected from parents/caregivers via the forms.

### 2.2 Collection and Weighing of Packed Lunches

Data collection was completed between April and July 2024. Data were collected over five consecutive days at each EYS and only from children whose parents/caregivers had provide consent. As children's attendance patterns varied and the number of children differed each day across the week, data were recorded from all consenting children on every day that child had a lunch at the EYS. Each child was identified by an ID number, and children's names were deleted once data collection was completed.

Before lunchtime, every item in each child's packed lunch was weighed. A new, clean container and pair of food‐grade gloves was used for each lunch and researchers wore protective clothing and hair nets to avoid contamination or transference of allergens between lunch boxes. All items were weighed to the nearest 1 g using Salter® scales. Individual pre‐packaged items (e.g., packets of crisps) were weighed, including the packaging weight. Items wrapped in foil or clingfilm were weighed in their wrapping. Items in bags or boxes were removed from their original container and weighed in a clean container where possible. Items that could not be removed from a container (e.g., pasta with cheese layered on top) were weighed within the container. Whole fruit and vegetables were weighed including skin and core/seeds. A full description of each food item was recorded to support later coding and analysis.

EYS staff were asked to retain packaging if this would normally be disposed of, and lunches were observed by the researcher to support this. After children had finished eating, leftover food was collected and weighed. Empty leftover packaging (wrappers, foil and clingfilm), boxes and bags were also weighed. For each food item, the total provided weight (including packaging), packaging weight, food weight (weight of the food as served, without packaging), and leftover food weight was recorded.

### 2.3 Coding of Food and Packaging

Data were entered into Excel. Edible portion weights were calculated (e.g., for fruit with peel) using standard estimates [25], and the amount of each food consumed was calculated from edible portion weights and leftover weights. Each item was also coded to record the level of packaging. Items were coded as either ‘individual pre‐packaged items’ (e.g., packets of crisps, boxes of raisins, pots or tubes of yoghurt), ‘other packaged items’ (purchased foods removed from original packaging, e.g., a sausage roll removed from its multipack), ‘unpackaged food items’ (e.g., whole pieces of fruit), ‘prepared food items’ (prepared single food items, e.g., vegetable slices or a boiled egg) or ‘composite food items’ (more than one food item combined, e.g., homemade sandwiches, pasta salads).

### 2.4 Analysis of Nutrient Content and Food Processing

Nutritional analysis of the weighed packed lunches was conducted using Nutritics® software [26]. Each packed lunch was entered as a separate log using the recorded item descriptions, edible portion weights (for analysis of the lunches as provided) and portion weights consumed (for analysis of the lunches as consumed). Food codes were chosen based on data from the McCance and Widdowson data set [25], and use of standard codes for common food items were agreed to ensure consistency. Where existing food codes were not a suitable match for products provided, new foods were inputted using nutritional data (per 100 g) from the manufacturer or retailer website, with the closest standard match used for micronutrient content to avoid underestimation. Sandwiches were entered as recipes, using the food descriptions (e.g., medium sliced wholemeal bread with spread and cheddar cheese) and standard food weights to estimate the proportion of different ingredients. Where other composite dishes were provided (e.g., pasta and sauce), a close‐matching recipe was obtained (e.g., from recipes included in McCance and Widdowson or the BBC food website [25, 27]) for entry, and cooking methods applied where appropriate to adjust for nutrient losses. Queries regarding missing or unclear information were resolved by consensus and entry from 10% of packed lunches was checked to ensure consistency and reliability. As differing numbers of packed lunches were weighed for individual children, the energy and nutrient content of an average packed lunch was calculated for each child, using the mean for lunches weighed across the week. Energy and nutrient content of lunches were compared to the nutrient framework for lunches underpinning the ‘Eat Better, Start Better’ voluntary food and drink guidelines for Early Years Settings in England, calculated based on dietary reference values for children aged 1–4 years [10]. Nutrients included in the framework as a minimum or maximum were classed as ‘met’ if mean content was at least, or no more than, the stated value respectively. Nutrients stating ‘approx’ (energy, fat and carbohydrate) were classed as ‘met’ if mean content was within 5% of the stated value.

Data were also coded to record the level of processing of each food item and ingredient. Coding was completed by one researcher using recorded information on food type, description and brand, and was based on the NOVA food classification system [28]. Foods were each coded as one of four groups defined according to the extent and purpose of food processing. Group 1 (unprocessed or minimally processed foods) included foods such as fresh fruit and vegetables, eggs, plain milk; Group 2 (processed culinary ingredients) included oils, sugars, and salt; Group 3 (processed foods) included canned fish, cream cheese and cheddar cheese; Group 4 (ultra‐processed foods) included sliced bread, flavoured yoghurts/fromage frais, crisps, biscuits, processed fruit bars and meat products such as sausage rolls. Foods not listed as examples within Monteiro et al. [28]. were coded based on classification within open food facts data and/or reference to previous research [29, 30]. Coding was checked by the second researcher and queries resolved by consensus.

### 2.5 Analysis of Foods Within Packed Lunches

Each packed lunch was coded to indicate the presence or absence of specific foods and food groups as recommended by ‘Eat Better, Start Better’ [10]. Recommended food groups included: starchy foods, fruit, vegetables, non‐dairy protein and dairy or alternatives. Foods recommended to limit or avoid included: meat products, processed fruit bars/cereal bars, savoury/composite snacks, cakes/biscuits, chocolate/confectionery and squash/cordial. A score out of 11 was calculated for each packed lunch to indicate how many of the recommendations above were met. Lunches were then divided into ‘healthier’ lunches (meeting eight or more of the 11 recommendations) and ‘less healthy lunches’ (meeting seven or fewer of the 11 recommendations).

### 2.6 Analysis of Packed Lunch Costs

The cost of each individual item (per 100 g/100 ml/item) was recorded in July 2024. For staple unbranded foods (e.g., whole fruit/vegetables, bread, ham, cheese) the Tesco website (https://www.tesco.com/groceries) was used to obtain cost information, as the supermarket with the highest market share, and the cheapest of the main (non‐discount) supermarkets in June 2024 [31, 32]. The cost of the cheapest available own brand option was recorded for each food and adjusted to calculate the cost per 100 g of edible food where applicable. Where products were on offer, the offer price was applied. Where foods provided were of a specific brand or from a different supermarket, the costs of these specific products were used.

### 2.7 Analysis by Area Deprivation

The postcode of each EYS was used to determine area deprivation using the English Index of Multiple Deprivation (IMD) 2019 data [33], and the deprivation decile for the lower‐layer super output area into which each postcode was located was recorded. EYS were then classed as ‘higher deprivation’ (IMD 1–5) or ‘lower deprivation’ (IMD 6–10).

### 2.8 Statistical Analysis

Data on energy, nutrient and cost of packed lunches were not normally distributed in most cases and are displayed as median values, with mean values also provided to enable comparison with previous studies and nutrient frameworks. For categorical data, Fisher's exact tests were used. An independent samples t‐test was used where data was normally distributed with Mann–Whitney *U* tests where data were not normally distributed. A value of *p* < 0.05 was used to describe statistical significance in all cases.

## 3 Results

Eight EYS were recruited to take part in the research (two schools, three private nurseries and three community nurseries/pre‐schools). Children in five EYS could eat a setting‐provided lunch or bring a packed lunch from home and in three EYS were required to bring a packed lunch. A weighed lunchtime intake was recorded from 115 participants. Four children were excluded from the analysis (one participant was aged 5, two were aged 2 years, and one was observed sharing their lunch items with other children). Of the 111 remaining participants, 67 had a packed lunch on one or more days of data collection. Data on setting‐provided lunches are reported separately.

Two children ate foods from their packed lunch and foods provided by the setting, during the same lunchtime (as foods from the setting lunches were given to supplement their packed lunches or for them to try). They remained in the analysis but were excluded on the day when they also consumed setting‐provided lunch items. A total of 185 individual packed lunches were included. The mean and median number of lunches were 2.8 and 3.0 respectively, per child (Table 1). Slightly more boys (55%) and older children (aged 4, 67%) were included in the study. Four settings were situated in areas of higher deprivation (deciles 1–5, 50%) and four were within areas of lower deprivation (deciles 6–10, 50%) (Table 1).

**Table 1:** Participant and setting characteristics.

Participant characteristics	*n* (%)
Boys	37 (55%)
Girls	30 (45%)
Aged 3 years	22 (33%)
Aged 4 years	45 (67%)
Total number of packed lunches observed	185
Mean no. of packed lunches per child (standard deviation)	2.8 (1.2)
Median no. of packed lunches per child (interquartile range) Range of packed lunches per child	3.0 [1–5] 1–5

Setting characteristics	***n* (%)**
Settings	8 (100%)
Settings where PL were consumed	8 (100%)
Settings where only PL were consumed (no setting‐provided lunch was available)	3 (37.5%)
Setting type	
School‐based nursery/pre‐school	2 (25%)
Private day nursery	3 (37.5%)
Voluntary/community nursery	3 (37.5%)
Area deprivation	
Higher (IMD deciles 1–5)	4 (50%)
Lower (IMD deciles 6–10)	4 (50%)

### 3.1 Packed Lunch Costs

The median cost of a packed lunch was £1.26 (mean £1.42) (Table 2). Packed lunches were of similar cost, whether provided to girls or boys (median £1.36 and £1.23 respectively, *p* = 0.407), children aged 3 or 4 (median £1.26 or £1.25 respectively, *p* = 0.585) or whether they were provided in areas of higher or lower deprivation (median £1.25 or £1.27 respectively, *p* = 0.422). Lunches with a higher healthy eating score (£1.23) were significantly cheaper than lunches with a lower healthy eating score (£1.72) (*p* < 0.001).

**Table 2:** The cost of a packed lunch.

Cost of packed lunches	*n*	Mean	SD	Median	(IQR)	Range (cheapest to most expensive)
Cost of a packed lunch						
All lunches	185	1.42	(0.59)	1.26	(0.82–1.70)	0.34–4.14
Provided for girls	84	1.48	(0.65)	1.36	(0.92–1.70)	0.34–4.14
Provided for boys	101	1.38	(0.53)	1.23	(0.83–1.63)	0.34–2.70
Provided for 3‐year‐olds	67	1.39	(0.56)	1.26	(0.80–1.72)	0.34–2.70
Provided for 4‐year‐olds	118	1.45	(0.61)	1.25	(0.83–1.69)	0.34–4.14
Deprivation						
Higher (IMD deciles 1–5)	57	1.51	(0.65)	1.25	(0.69–1.82)	0.34–3.00
Lower (IMD deciles 6–10)	128	1.39	(0.56)	1.27	(0.64–1.64)	0.34–4.14
Healthy eating score						
Lower (0‐7)	69	1.62	(0.58)	1.72	(1.22–2.22)	0.56–2.86*
Higher (8–11)	116	1.31	(0.56)	1.23	(0.91–1.55)	0.34–4.14

Abbreviations: IQR, Inter‐quartile range; SD, standard deviation.

* Denotes a statistical difference between groups, *p* < 0.05 (Mann–Whitney *U* test).

### 3.2 Food Items Provided

**Figure 1:**
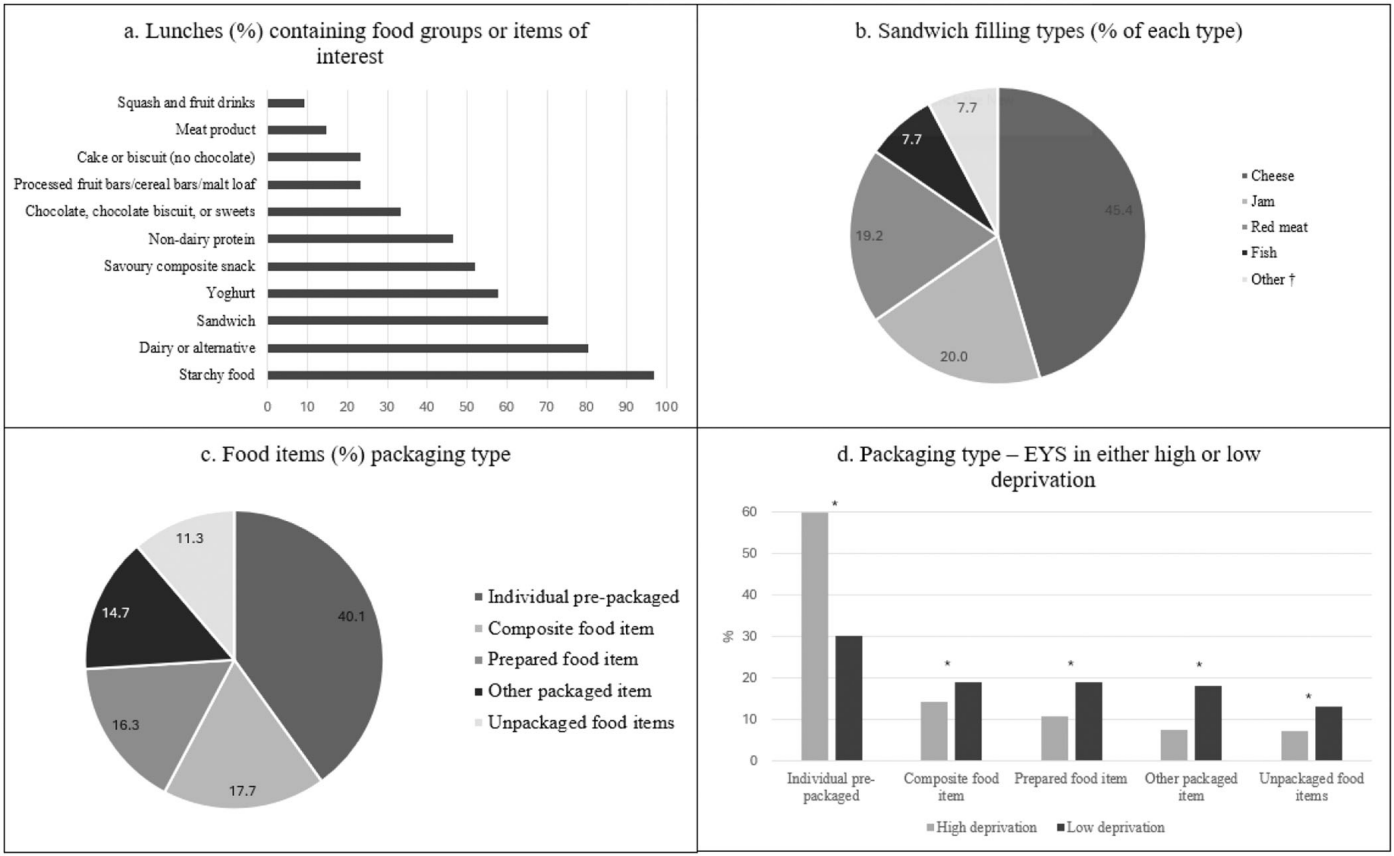
Foods, sandwich fillings and packaging types provided to all children and to children attending early years settings (EYS) in varying levels of deprivation. †Other sandwich fillings included hummus, chicken, chocolate spread, egg and peanut butter. *Denotes a statistical difference in packaging between high deprivation and low deprivation, *p* < 0.05 (Fisher's exact test). a. Luches (%) containing food groups or items of interest. b. Sandwich filling types (% of each type). c. Food items (%) packaging type. d. Packaging type – EYS in either high or low deprivation.

Packed lunches contained a variety of foods items (Figure 1a and Table 3). Almost all lunches (96.8%) included a starchy food, 80.5% included a dairy or alternative food, 76.2% included fruit, 46.5% included a non‐dairy protein and 38.9% included vegetables (Table 3). Packed lunches in more deprived areas were, however, less likely to contain fruit (61.4% compared to 82.8%, *p* = 0.003), vegetables (10.5% compared to 51.6%, *p* < 0.001), a portion of fruit (57.9% compared to 77.3%, *p* = 0.009) or a portion of vegetables (3.5% compared to 36.2%, *p* < 0.001) (Table 3). Packed lunches in areas of higher deprivation were also more likely to contain meat products (33.3% vs. 6.3%, *p* < 0.001), savoury/composite savoury snacks (75.4% compared to 41.4%, *p* < 0.001), cakes and biscuits (33.3% compared to 18.8%, *p* = 0.038), chocolate or sugar confectionary (or foods containing these) (64.9% compared to 19.5%, *p* < 0.001), and squash/fruit drinks (19.3% compared to 4.7%, *p* = 0.004) (Table 3). Around 70% of lunches included a sandwich. The most frequently provided sandwich fillings were cheese (45.4%), jam (20.0%), red meat (predominantly ham) (19.2%) and fish (7.7%) (Figure 1b). Lunches had a median healthy eating score of 8.0, with a higher median healthy eating score in areas of low deprivation (8.5) compared with high deprivation (6.4) (*p* < 0.001).

**Table 3:** Food items provided as part of a packed lunch.

Food item or group	Percentage of lunches containing this food item			*p* value^a^
	Overall (*n* = 185)	More deprived (*n* = 57)	Less deprived (*n* = 128)	
Fruit	76.2	61.4	82.8	0.003*
A portion of fruit	71.4	57.9	77.3	0.009*
Vegetables	38.9	10.5	51.6	< 0.001*
A portion of vegetables	26.1	3.5	36.2	< 0.001*
Starchy food	96.8	93.0	98.4	0.740
Non‐dairy protein	46.5	47.4	46.1	0.875
Dairy or alternative	80.5	77.2	82.0	0.430
Yoghurt	57.8	64.9	54.7	0.202
Sandwiches	70.3	75.4	68.0	0.384
Meat products	14.6	33.3	6.3	< 0.001*
Processed fruit or cereal bar (includes malt loaf)	23.2	19.3	25.0	0.455
Savoury snack or composite savoury snack	51.9	75.4	41.4	< 0.001*
Cake or biscuit	23.2	33.3	18.8	0.038*
Chocolate or sugar confectionery	33.5	64.9	19.5	< 0.001*
Squash or fruit drinks	9.2	19.3	4.7	0.004*

^a^ *p* values calculated using Fisher's exact tests.

* Indicates statistically significant difference between more deprived and less deprived areas (*p* < 0.05).

### 3.3 Packaging and Extent of Processing

**Figure 2:**
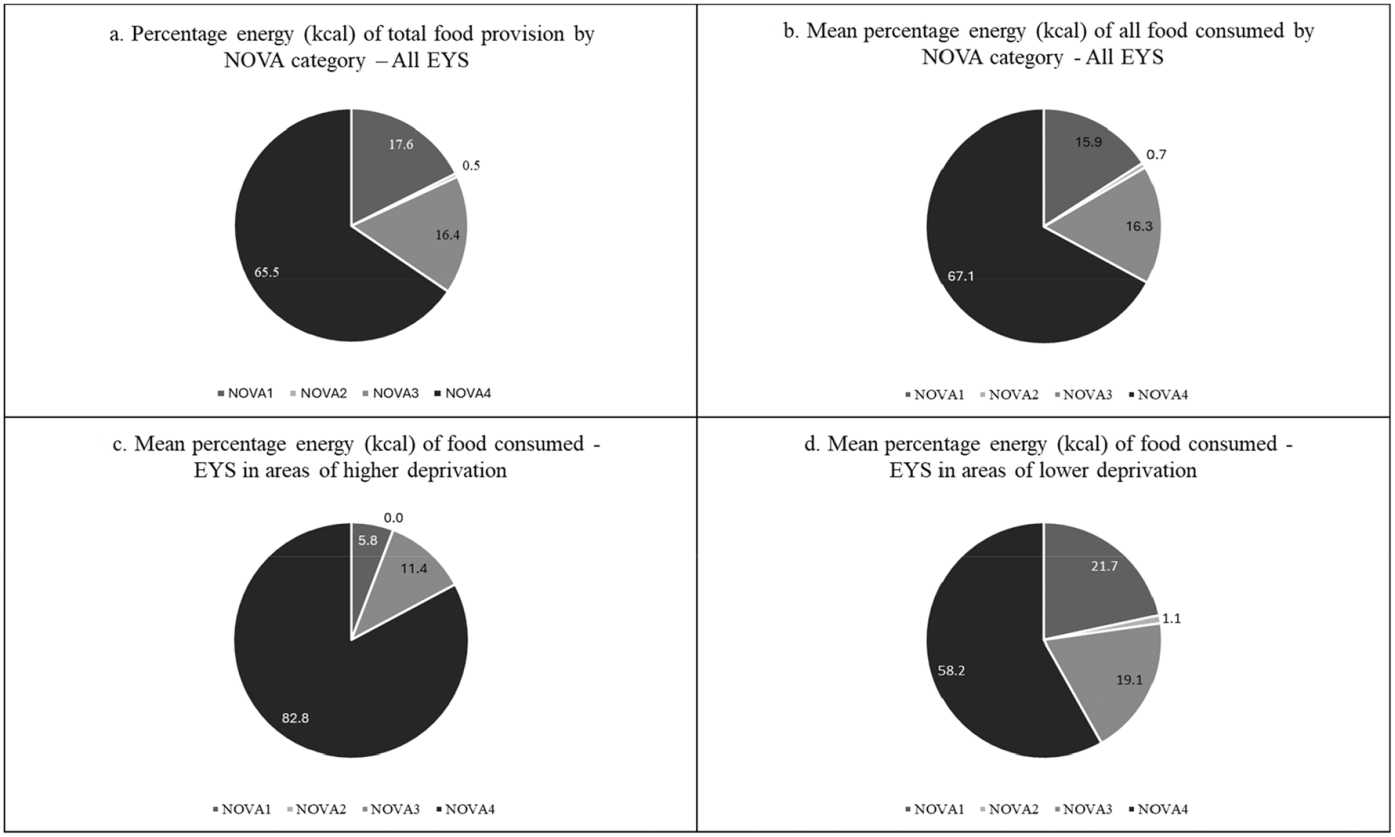
The percentage of energy provided and consumed by children attending early years settings (EYS) (all settings) and percentage of energy (kcal) as consumed by children attending EYS in areas of varying deprivation. a. Percentage energy (kcal) of total food provision by NOVA category ‐ All EYS. b. Mean percentage energy (kcal) of all food consumed by NOVA category – All EYS. c. Mean percentage energy (kcal) of food consumed ‐ EYS in areas of higher deprivation. d. Mean percentage energy (kcal) of food consumed ‐ EYS in areas of lower deprivation.

Across all lunches, the proportion of individually pre‐packaged items provided (40.1%), was higher than the proportion of composite items (17.7%), single prepared items (16.3%), other packaged items (14.7%) or unpackaged items (11.3%) (Figure 1c). Children attending EYS in areas of higher deprivation were provided with a higher proportion of individual pre‐packaged items (59.9% compared to 30.4%, *p* < 0.001) and fewer composite (14.5% *vs.* 19.2%, *p* = 0.036), prepared (10.8% vs. 19.0%, *p* < 0.001), other packaged (7.6% vs. 18.1%, *p* < 0.001) or unpackaged items (7.3% vs. 13.3%, *p* = 0.002) (Figure 1d). Yoghurts were included in 57.8% of lunches. These were often in pots (39.3%) but were also provided as tubes (38.3%), pouches (18.7%) or bottles (3.7%) that children could consume without a spoon. Ultra‐processed foods (NOVA 4 group) provided 65.5% of the energy provided for children, and 67.1% of energy consumed by children in the study (Figure 2a,b). Children attending EYS in areas of higher deprivation consumed significantly less energy from NOVA 1 (*p* < 0.001), NOVA 2 (*p* = 0.037) and NOVA 3 (*p* < 0.056) and more energy from NOVA 4 (82.8% vs. 58.2%) (*p* < 0.001) (Figure 2c,d).

### 3.4 Nutrient Content of Lunches as Provided and Consumed

On average, children were provided with food that met lunchtime nutrient recommendations [10, 34] for fibre, vitamin A, vitamin C, calcium, iodine, iron and zinc, regardless of area deprivation for the EYS. Median provision of energy, free sugars, sodium, protein, carbohydrate, fat and saturated fat were above recommended levels [10].

Children in areas of higher deprivation were provided with significantly more energy (median 584 kcal vs. 479 kcal, *p* < 0.001), carbohydrate (83.8 g vs. 58.6 g, *p* = 0.005), free sugars (23.4 g vs. 10.4 g, *p* < 0.001), fat (24.4 g vs. 18.4 g, *p* = 0.007), saturated fat (9.8 g vs. 7.3 g, *p* = 0.032) and sodium (746 mg vs. 559 mg *p* = 0.016) and less fibre (5.4 g vs. 6.3 g, *p* = 0.033) and vitamin C (6.6 mg vs. 26.2 mg, *p* = 0.029) per lunch than children attending EYS in areas of lower deprivation (Table 4).

**Table 4:** Nutrient content of packed lunches provided to children in early years settings.

Nutrient	Nutrient framework for lunches^b^	All participants (*n* = 67)				Higher deprivation (*n* = 22)				Lower deprivation (*n* = 45)				*p* value
		Mean	SD	Median	Interquartile range	Mean	SD	Median	Interquartile range	Mean	SD	Median	Interquartile range	
Energy (kJ)	~ 1542	2202	656	2171	1807–2536	2627	793	2451	1982–2920	1994	459	2023	1781–2266	< 0.001*
Energy (kcal)	~ 369	524	156	517	431–604	625	189	584	474–695	475	110	479	421–537	< 0.001*
Carbohydrate (g)	~ 49.2	68.0	24.5	63.8	45.8–81.8	82.2	30.8	83.8	63.8–103.8	61.0	17.3	58.6	45.5–71.8	0.005*
Free sugars (g)	≤ 4.9	16.4	13.0	13.5	5.4–21.7	25.9	15.4	23.4	14.4–32.4	11.7	8.4	10.4	5.3–15.6	< 0.001*
Fibre (g)	≥ 4.5	6.0	2.0	6.1	4.8–7.5	5.3	2.1	5.4	3.6–7.3	6.4	1.8	6.3	5.1–7.6	0.033^a^
Protein (g)	≥ 5.1	18.5	7.5	18.2	14.5–21.9	19.6	11.1	17.7	12.2–23.3	18.0	4.9	18.2	14.7–21.7	0.957
Fat (g)	~ 14.4	19.6	8.2	20.0	14.8–25.2	24.1	10.0	24.4	17.8–31.1	17.4	6.3	18.4	14.0–22.8	.007* ^,^ a
Saturated fat^c^ (g)	≤ 4.1	8.1	4.0	8.1	5.1–11.2	9.6	4.5	9.8	6.8–12.9	7.4	3.5	7.3	4.8–9.9	0.032* ^,^ a
Vitamin A (µg)	≥ 136	212	264	153	81–225	204	322	93	8–177	215	236	174	101–247	0.274
Vitamin C (mg)	≥ 9	24.4	20.7	22.2	6.0–38.5	20.7	27.4	6.6	−13–26.2	26.3	16.6	26.2	11.4–41.0	0.029* ^,^ a
Calcium (mg)	≥ 120	325	149	317	214–420	343	167	349	220–479	316	140	302	212–392	0.495^a^
Iodine^c^ (µg)	≥ 26	30	21	24	14–35	26	18	24	18–30	32	21	28	15–42	0.350
Iron (mg)	≥ 2.3	2.4	0.8	2.3	1.8–2.9	2.6	0.9	2.5	1.7–3.3	2.3	0.7	2.2	1.8–2.7	0.120
Zinc (mg)	≥ 1.7	2.1	0.7	2.1	1.6–2.7	2.1	0.8	1.8	1.1–2.5	2.2	0.7	2.2	1.8–2.7	0.577^a^
Sodium (mg)	≤ 300	653	307	604	433–776	815	408	746	472–1021	573	206	559	431–688	0.016* ^,^ a

*Note: p* values relate to comparison between energy/nutrient content of lunches provided for children attending settings in areas of higher and lower deprivation. *p* values are from Mann–Whitney *U* tests (variables not normally distributed) unless indicated otherwise.

Abbreviation: SD, standard deviation.

^a^ *p* values are from independent samples T test (variables are normally distributed).

^b^ The nutrient framework for lunches is from the voluntary food and drink guidelines for Early Years Settings in England and represents nutrient‐based standards for lunches for children aged 1–4 years attending early years settings [10].

^c^ Saturated fat and iodine are not included in the nutrient framework underpinning the voluntary food and drink guidelines but are included here due to concerns about intakes in young children. These have been calculated from UK dietary reference values (saturated fat to provide ≤ 10% energy; iodine to provide ≥ 30% of the RNI for children aged 3‐4 years in line with the method of calculation for other micronutrients within the nutrient framework) [32].

* Denotes significance (*p* value < 0.05).

Nutrient consumption was in line with recommendations for vitamin A, vitamin C and calcium for both groups and additionally in line with recommendations for energy, carbohydrate, fibre, fat, iodine and zinc amongst children attending EYS in areas of lower deprivation. Despite differences in provision and degree of adherence to recommendations, there were very few significant differences in intake between groups, with children attending EYS in areas of higher deprivation only consuming significantly more free sugars (median 17.7 g vs. 9.3 g, *p* = 0.002) and significantly less fibre (3.4 g vs. 4.8 g, *p* = 0.003) and vitamin C (2.0 g, vs. 18.0 g, *p* = 0.003) than children attending EYS in areas of lower deprivation (Table 5).

**Table 5:** Nutrient content of packed lunches consumed by children in early years settings.

Nutrient	Nutrient framework for lunches^b^	All participants (*n* = 67)				High deprivation (*n* = 22)				Low deprivation (*n* = 45)				*p* value
		Mean	SD	Median	Interquartile range	Mean	SD	Median	Interquartile range	Mean	SD	Median	Interquartile range	
Energy (kJ)	~ 1542	1750	588	1694	1359–2029	1940	822	1792	1101–2483	1657	411	1656	1387–1925	0.169
Energy (kcal)	~ 369	416	140	404	323–485	462	195	425	260–591	394	98	394	329–459	0.165
Carbohydrate (g)	~ 49.2	54.2	22.4	50.7	39.2–62.2	62.2	32.7	55.7	35.5–76.0	50.3	14.0	49.4	41.9–57.9	0.112
Free sugars (g)	≤ 4.9	13.3	11.4	10.4	4.7–16.1	20.7	15.4	17.7	8.3–27.1	9.8	6.6	9.3	4.9–13.7	0.002*
Fibre (g)	≥ 4.5	4.7	1.8	4.4	3.0–5.8	3.7	1.9	3.4	2.1–4.7	5.1	1.6	4.8	3.6–6.1	0.003^a^^,^ *
Protein (g)	≥ 5.1	15.2	6.3	14.4	10.3–18.6	14.8	8.6	12.9	8.5–17.4	15.4	4.9	15.1	11.3–19.0	0.400
Fat (g)	~ 14.4	15.3	7.1	13.6	8.8–18.5	17.0	8.9	13.6	7.3–19.9	14.4	6.0	14.5	10.5–18.6	0.238^a^
Saturated fat^c^ (g)	≤ 4.1	6.3	3.6	5.7	3.3–8.2	6.7	4.0	5.6	2.8–8.5	6.2	3.4	5.7	3.3–8.2	0.599^a^
Vitamin A (µg)	≥ 136	159	186	128	61–195	143	237	81	4–159	167	157	134	75–193	0.093
Vitamin C (mg)	≥ 9	18.6	18.3	12.1	−0.2–24.4	13.6	21.0	2.0	‐9.7–13.7	21.0	16.6	18	5–31	0.003*
Calcium (mg)	≥ 120	269	143	242	154–330	260	153	225	132–319	274	138	261	174–349	0.566
Iodine^c^ (µg)	≥ 26	26	20	21	8–33	20	18	14	5–23	28	20	23	11–35	0.067
Iron (mg)	≥ 2.3	1.9	0.8	1.8	1.4–2.3	2.0	1.0	1.8	1.2–2.5	1.9	0.7	1.8	1.4–2.3	0.862
Zinc (mg)	≥ 1.7	1.8	0.7	1.6	1.1–2.2	1.6	0.8	1.3	0.6–2.0	1.8	0.6	1.8	1.4–2.3	0.122^a^
Sodium (mg)	≤ 300	522	251	457	317–598	611	317	529	314–745	478	201	427	302–552	0.063

*Note: p* values relate to comparison between energy/nutrient content of lunches provided for children attending settings in areas of higher and lower deprivation. *p* values are from Mann–Whitney *U* tests (variables not normally distributed) unless indicated otherwise.

Abbreviation: SD, standard deviation.

* *p* < 0.05.

^a^ *p* values are from independent samples T test (variables are normally distributed).

^b^ The nutrient framework for lunches is from the voluntary food and drink guidelines for Early Years Settings in England and represents nutrient‐based standards for lunches for children aged one to four years attending early years settings [10].

^c^ Saturated fat and iodine are not included in the nutrient framework underpinning the voluntary food and drink guidelines but are included here due to concerns about intakes in young children. These have been calculated from UK dietary reference values (saturated fat to provide ≤ 10% energy; iodine to provide ≥ 30% of the RNI for children aged 3–4 years in line with the method of calculation for other micronutrients within the nutrient framework) [32].

The proportion of nutrients consumed from available provision was also explored. Children in areas of lower deprivation consumed significantly more (80.2%–86.8% compared to 61.0%–74.6%) of the nutrients provided, for every nutrient except carbohydrate, fibre, vitamin A, sodium and iron. Consumption of provided vitamin C was particularly low amongst children in higher deprivation areas (61.0% of provided vitamin C consumed) (Table 6).

**Table 6:** Proportion of food and nutrients, consumed by children.

		Percentage of each nutrient consumed		
Nutrient	All children (*n* = 67)	Higher deprivation (*n* = 22)	Lower deprivation (*n* = 45)	*p* value
Energy (kJ)	80.4	72.6	84.3	0.037*
Energy (kcal)	80.4	72.6	84.2	0.035*
Carbohydrate (g)	80.8	74.6	83.8	0.200
Free sugars (g)	82.4	74.4	86.4	0.026*
Fibre (g)	78.6	72.4	81.6	0.214
Protein (g)	82.6	74.9	86.3	0.031*
Fat (g)	78.9	68.6	84.0	0.008*
Saturated fat (g)	78.8	68.8	83.7	0.013*
Vitamin A (µg)	77.5	69.4	81.4	0.084
Vitamin C (mg)	73.9	61.0	80.2	0.025*
Calcium (mg)	82.1	73.5	86.3	0.013*
Iodine (µg)	81.4	70.4	86.8	0.007*
Iron (mg)	81.0	75.3	83.8	0.121
Zinc (mg)	82.1	74.3	85.9	0.022*
Sodium (mg)	81.2	74.7	84.4	0.040*

* Denotes a statistically significant difference between higher deprivation and lower deprivation, *p* < 0.05 (Mann–Whitney *U* tests).

## 4 Discussion

This study aimed to estimate the energy and nutrient content of packed lunches provided for, and consumed by, children attending EYS. Also, the cost of lunches, level of processing and types of food provided, and whether this differed by area deprivation.

The estimated median and mean energy content of packed lunches as provided (517/524 kcal), was higher than recommended for lunches in the ‘Eat Better, Start Better’ guidance (~369 kcal) and was also greater than mean provision in the 2012 pre‐school food survey (442 kcal), with significantly more energy provided to children attending EYS in areas of higher deprivation than in areas of lower deprivation [10, 11]. Our results are also consistent with those from studies that have considered provision for pre‐school and primary school children, in finding fat, saturated fat, sugars and sodium were higher than recommended [10, 11, 35].

In part, excess energy, fat, sugars and sodium were due to the frequent provision of energy dense foods observed in the study, such as meat products, savoury snacks, cakes and biscuits, and chocolate and confectionery, particularly to children attending EYS in areas of higher deprivation. Children were provided with these items less often than in studies from primary schools (51.9% compared to 81% of lunches containing savoury snacks, 23.2% vs. 81% containing cakes and biscuits and 33.5% compared to 86% being provided with confectionery‐containing foods) [35] with the proportion of lunches containing these items similar to the pre‐school food survey [11]. Overall provision of vegetables was low (38.9%) but higher than in studies carried out in primary schools (19%–20%) [35] and previously in EYS (< 20%) [11]. Provision of fruit was also higher (71.4% of lunches), compared to 53.6%–57% in schools [35, 36].

Provision clearly exceeded children's requirements, and practitioners commonly reported that they believed parents provide a range of foods for children to choose from, knowing they won't all be consumed. Despite differences in the amounts and types of food provided to children attending EYS in areas of varying deprivation, intakes of energy and key nutrients differed little between groups. Average consumption by children in areas of both higher and lower deprivation contained amounts of vitamin A, vitamin C and calcium in line with recommendations.

Children attending EYS in areas of lower deprivation also met guidelines for intake of energy, carbohydrate, fibre, fat, iodine, and zinc, possibly due to more frequent consumption of fruit, vegetables, meat and fish. Intakes of vitamin C and fibre were also higher for these children, due to more frequently provided and larger portions (> 40 g) of fruit and vegetables and a higher percentage consumption of the fruit and vegetables provided. Meanwhile, children attending EYS in areas of higher deprivation consumed significantly more free sugars (almost four times the maximum amount recommended, and providing 16.8% of energy intake) double the amount consumed by children attending EYS in areas of lower deprivation (9.3% of energy) [10, 37]. The leading contributors to free sugars intake in children's diets are well established (yoghurt/fromage frais, biscuits, fruit juice, cakes and confectionery) and include many foods typically found in packed lunches [3, 38, 39]. All previous studies have shown packed lunches to be high in sugars, but it was disappointing that levels have not reduced since 2013 when lunches provided 16.7 g sugars in comparison with 16.4 g in this study, given the public health messaging and interventions around sugar reduction [39, 40, 41]. As well as frequent provision of cakes and biscuits, confectionery‐containing items and processed fruit bars, 20% of the sandwiches observed in the study contained jam, higher than the proportion of sweet sandwich fillings seen in previous research [42].

Protein provision and consumption were both high in this study, with children consuming most of their daily protein requirement (14.5 g for 3‐year‐olds and 19.5 g for 4‐year‐olds) at lunchtime [34]. This is consistent with the pre‐school food survey, our previous research on food provision to children attending school nurseries, and with UK‐wide surveys that suggest protein intake is higher than recommended in young children [11, 38, 39, 43, 44]. Higher protein intakes may be associated with a higher body mass index in childhood, and SACN recently recommended that the UK government should focus on reducing intakes in young children [3].

UPF (NOVA 4) made up 65.5% of the energy content of food provided across all lunches, with a significantly higher proportion of energy provided by UPF in EYS in areas of higher deprivation (81.0%) compared to those in areas of lower deprivation (58.7%). The overall contribution of UPF to energy intake is higher (67.1%) than the 46.9% found by Conway et al. [45] in younger toddlers, but more aligned to the 61% estimated for 2–5‐year‐old UK children by Neri et al. [46] based on NDNS data. Our results may be higher than Conway et al., as the children in our study were older (UPF intake appears to increase during childhood) [45, 46]. Packed lunches are also likely to contain a higher proportion of UPF than found in the total diet—40% of the items recorded in our study were purchased individually pre‐packed items convenient for lunch boxes (e.g., crisps, processed fruit bars, yoghurts and confectionery items), all of which are ultra‐processed. Finally, bread is a staple of packed lunches and commercially available bread is largely classed as UPF. Parnham et al. [47] found a higher overall proportion of energy intake from UPF (81.2%) in their study of primary school packed lunches and as with our study, reported associations between higher UPF content and IMD, with higher consumption amongst children in more deprived areas. Our study adds to a growing body of evidence highlighting concerns about the impact of high UPF consumption on the nutritional quality of children's diets [45, 46].

The median cost of a packed lunch provided in the study was £1.26. There was no difference in cost of lunches in areas of higher and lower deprivation. Lunches in areas of lower deprivation contained more fruit and vegetables, but the higher cost of these foods appeared to be offset by the higher cost of the individually pre‐packed items more commonly provided in areas of higher deprivation. This may indicate that saving time is more important than cost when planning packed lunches, with pre‐packaged foods offering a quick and easy alternative to washing, preparing and cooking composite dishes or fresh fruit and vegetables [48]. Healthier lunches were also cheaper (median cost £1.22) than less healthy lunches (median cost £1.72) again reflecting differences in the types of food provided in less healthy lunches. This aligns with recent research from Australia that suggested higher packed lunch costs were predicted by a higher proportion of energy from unhealthy foods and higher levels of socioeconomic disadvantage [21] but differs from a recent cost analysis of indicative packed lunches (designed for older children) which estimated healthier packed lunches may be 45% more expensive [22]. Together these findings suggest families require cheap convenient items that are minimally processed and nutritious if the nutritional quality of packed lunches are to be improved.

This study provides much needed evidence regarding packed lunch provision and consumption within EYS. It is the first to use weighed intake methodology and prospectively collect data to be coded using NOVA [28], and also provides cost estimates of lunches. There are, however, several limitations when interpreting the findings of the study. Research was conducted in eight EYS in Sheffield, and although the use of purposive sampling in recruitment ensured variation in setting type and area deprivation, findings may not be reflective of EYS locally or nationally. Those with better practices or more interest in food may have been more likely to participate. Although recruitment aimed to reflect varying area deprivation, EYS were all located in IMD deciles 1–2 and 6–10 and representation of settings in decile 3–5 was missing. Although most settings mentioned sharing information or guidance with parents on healthy packed lunches, the content of and engagement with this was not recorded as part of the study. Furthermore, lunches were only analysed where parental consent was received and lunches of children where consent was provided may differ from those where it was not. Children taking a packed lunch may also differ from those who eat a setting lunch where setting lunches are provided (e.g., due to fussy eating, special educational needs, or socioeconomic status (where the cost of setting‐provided lunches may be prohibitive)). Parents were not informed of when data collection would occur to reduce the risk of conscious changes to foods provided for social desirability reasons, but as data collection occurred over a week, children may have told their parents that data collection had started. Data collection was completed between April and July 2024, and some foods were clearly seasonal (e.g., strawberries). Costing of lunches was completed in July 2024, to ensure cost data was representative of the period in which lunches were provided, but branded items could have been purchased from cheaper or more expensive outlets. Recipes were not available for composite dishes such as pasta sauces and homemade cakes and the analysis of sandwiches was based on the recorded on overall weights and descriptions. The exact weight of each component was estimated.

## 5 Conclusion

Despite concerns over childhood obesity and a widespread interest in food provision within schools and EYS, packed lunches in this study were characterised by a high frequency of individually packaged, ultra‐processed, discretionary foods and low frequency of vegetables. Intakes of free sugars and sodium exceeded recommendations. Healthier lunches were cheaper than less healthy lunches and time may be the key constraining factor for many parents. Further support may be required for parents to help them prepare better quality packed lunches quickly, without increasing the cost.

## Author Contributions

Jo Pearce designed the study, collected data, performed analysis, and authored the paper. Claire J. Wall designed the study, collected data, performed analysis, and authored the paper.

## Ethics Statement

This study was conducted according to the guidelines laid down in the Declaration of Helsinki and all procedures involving research study participants were approved by the Sheffield Hallam University research ethics review system (ID:ER61543155).

## Consent

Written informed consent was obtained from all subjects.

## Conflicts of Interest

The authors declare no conflicts of interest.

### Peer Review

The peer review history for this article is available at https://www.webofscience.com/api/gateway/wos/peer-review/10.1111/jhn.70066.

## Transparency Declaration

The lead author affirms that this manuscript is an honest, accurate, and transparent account of the study being reported. The reporting of this study is compliant with STROBE guidelines. The lead author affirms that no important aspects of the study have been omitted and that any discrepancies from the study as planned have been explained.

## Data Availability

The data that support the findings of this study are available on request from the corresponding author. The data are not publicly available due to privacy or ethical restrictions.

## References

[1] C. Mameli , S. Mazzantini , and G. Zuccotti , “Nutrition in the First 1000 Days: The Origin of Childhood Obesity,” International Journal of Environmental Research and Public Health 13, no. 9 (2016): 838. 27563917 10.3390/ijerph13090838 PMC5036671

[2] M. Rolland‐Cachera , M. Akrout , and S. Péneau , “Nutrient Intakes in Early Life and Risk of Obesity,” International Journal of Environmental Research and Public Health 13, no. 6 (2016): 564–567. 27275827 10.3390/ijerph13060564 PMC4924021

[3] “Feeding Young Children Aged 1 to 5 Years,” Scientific Advisory Committee on Nutrition, 2023. 10.1017/S0029665126102250 41748544

[4] The Food Foundation , “The Food Foundation: Food Insecurity Tracking,” 2024, https://foodfoundation.org.uk/initiatives/food-insecurity-tracking#tabs/Round-15.

[5] V. Wight , N. Kaushal , J. Waldfogel , and I. Garfinkel , “Understanding the Link Between Poverty and Food Insecurity Among Children: Does the Definition of Poverty Matter?,” Journal of Children and Poverty 20, no. 1 (2014): 1–20. 25045244 10.1080/10796126.2014.891973 PMC4096937

[6] Department for Education , Education Provision: Children Under 5 Years of Age (Department for Education, 2024).

[7] M. Marmot , P. Goldblatt , and J. Allen , Fair Society, Healthy Lives: Strategic Review of Health Inequalities in England, Post‐2010 (Institute of Health Equity, 2010).

[8] L. Birch , J. S. Savage , and A. Ventura , “Influences on the Development of Children's Eating Behaviours: From Infancy to Adolescence,” Canadian Journal of Dietetic Practice and Research 68, no. 1 (2007): s1–s56. 19430591 PMC2678872

[9] S. Nicklaus , “The Role of Food Experiences During Early Childhood in Food Pleasure Learning,” Appetite 104 (2016): 3–9. 26298009 10.1016/j.appet.2015.08.022

[10] Action for Children , “Eat Better, Start Better. Voluntary Food and Drink Guidelines for Early Years Settings in England.” Action for Children (2017).

[11] J. Nicholas , L. Stevens , L. Briggs , and L. Wood , “Pre‐school Food Survey,” Children's Food Trust, 2013.

[12] E. Haney , J. C. Parnham , K. Chang , et al., “Dietary Quality Of School Meals And Packed Lunches: A National Study of Primary And Secondary School Children in the UK,” Public Health Nutrition 26, no. 2 (2023): 425–436. 35641314 10.1017/S1368980022001355 PMC13076075

[13] C. E. L. Evans , C. L. Cleghorn , D. C. Greenwood , and J. E. Cade , “A Comparison of British School Meals and Packed Lunches From 1990 to 2007: Meta‐analysis by Lunch Type,” British Journal of Nutrition 104, no. 4 (2010): 474–487. 20500928 10.1017/S0007114510001601

[14] J. Pearce , C. Harper , D. Haroun , L. Wood , and M. Nelson , “Short Communication Key Differences Between School Lunches and Packed Lunches in Primary Schools in England in 2009,” Public Health Nutrition 14, no. 8 (2011): 1507–1510. 21272423 10.1017/S1368980010003605

[15] J. Pearce and M. Nelson , “Comparison Between School Lunches and Packed Lunches in Secondary Schools,” Proceedings of the Nutrition Society 70 (2011): E168.

[16] L. Stevens and M. Nelson , “The Contribution of School Meals And Packed Lunch to Food Consumption and Nutrient Intakes in UK Primary School Children From a Low Income Population: Effect of Low Income On School Lunch Intakes,” Journal of Human Nutrition and Dietetics 24, no. 3 (2011): 223–232. 21332839 10.1111/j.1365-277X.2010.01148.x

[17] R. E. Conway , G. N. Heuchan , L. Heggie , et al., “Ultra‐Processed Food Intake in Toddlerhood and Mid‐childhood in the UK: Cross Sectional and Longitudinal Perspectives,” European Journal of Nutrition 63, no. 8 (2024): 3149–3160. 39363048 10.1007/s00394-024-03496-7 PMC11519182

[18] J. C. Parnham , K. Chang , F. Rauber , et al., “The Ultra‐Processed Food Content of School Meals and Packed Lunches in the United Kingdom,” Nutrients 14, no. 14 (2022): 2961–2975. 35889918 10.3390/nu14142961 PMC9318725

[19] G. A. Goodchild , J. Faulks , J. A. Swift , J. Mhesuria , P. Jethwa , and J. Pearce , “Factors Associated With Universal Infant Free School Meal Take Up and Refusal in A Multicultural Urban Community,” Journal of Human Nutrition and Dietetics 30, no. 4 (2017): 417–428. 28139045 10.1111/jhn.12442

[20] School Food Matters, Impact on Urban Health, Bremner & Co ., Cost of a School Meal (School Food Matters, 2024).

[21] A. C. Manson , B. J. Johnson , L. Wolfenden , R. Sutherland , and R. K. Golley , “Unpacking the Cost of the Lunchbox for Australian Families: A Secondary Analysis,” Health Promotion International 39, no. 1 (2024): daad19, 10.1093/heapro/daad194. PMC10781432 38198723

[22] The Food Foundation , “Healthy Packed Lunches Cost on Average 45% More Than Unhealthy Options,” The Food Foundation, 2024, https://foodfoundation.org.uk/sites/default/files/2024-05/KFG%20Packed%20lunches%20May.pdf.

[23] Ofsted , “Check If Childcare Is Registered,” 2024, https://reports.ofsted.gov.uk/childcare.

[24] UK Government , “Get Information About Schools,” 2024, https://www.get-information-schools.service.gov.uk/.

[25] R. A. McCance and E. M. Widdowson , McCance and Widdowson's the Composition of Foods (Cambridge: Royal Society of Chemistry, 2014).

[26] Nutritics , “Research Edition (v5.64),” 2022.

[27] BBC , “BBC Good Food,” 2024, https://www.bbcgoodfood.com.

[28] C. A. Monteiro , G. Cannon , M. Lawrence , M. L. da Costa Louzada , and P. Pereira Machado , Ultra‐Processed Foods, ‐ Diet Quality, and Health Using the NOVA Classification System (FAO, 2019).

[29] I. Y. Chavez‐Ugalde , F. de Vocht , R. Jago , et al., “Ultra‐Processed Food Consumption in Uk Adolescents: Distribution, Trends, And Sociodemographic Correlates Using the National Diet and Nutrition Survey 2008/09 to 2018/19,” European Journal of Nutrition 63, no. 7 (2024): 2709–2723. 39014218 10.1007/s00394-024-03458-z PMC11490440

[30] “Open Food Facts,” 2025, https://world.openfoodfacts.org/.

[31] Statista , “Market Share of Grocery Stores in Great Britain from January 2017 to April 2024,” 2024, https://www.statista.com/statistics/280208/grocery-market-share-in-the-united-kingdom-uk/.

[32] “Reviews: Cheapest Supermarkets,” Which?, 2024, https://www.which.co.uk/reviews/supermarkets/article/supermarket-price-comparison-aPpYp9j1MFin.

[33] “Ministry of Housing, Communities & Local Government. English Indices of Deprivation 2019,” IMD, 2024, https://imd-by-postcode.opendatacommunities.org/imd/2019.

[34] Department of Health , Dietary Reference Values for Food Energy and Nutrients for the United Kingdom (Department of Health, 1991).

[35] C. E. L. Evans , D. C. Greenwood , J. D. Thomas , and J. E. Cade , “A Cross‐Sectional Survey of Children's Packed Lunches in the UK: Food‐ and Nutrient‐Based Results,” Journal of Epidemiology & Community Health 64, no. 11 (2010): 977–983. 20089755 10.1136/jech.2008.085977

[36] C. E. L. Evans , K. E. Melia , H. L. Rippin , N. Hancock , and J. Cade , “A Repeated Cross‐Sectional Survey Assessing Changes in Diet and Nutrient Quality of English Primary School Children's Packed Lunches Between 2006 and 2016,” BMJ Open 10, no. 1 (2020): e029688. 10.1136/bmjopen-2019-029688 PMC7045752 31932386

[37] Scientific Advisory Committee on Nutrition , “Carbohydrates and Health. Scientific Advisory Committee on Nutrition,” 2015.

[38] A. Lennox , J. Sommerville , K. Ong , H. A. Henderson , and R. Allen , “Diet and Nutrition Survey of Infants and Young Children, 2011,” Department of Health and Food Standards Agency, 2013.

[39] Public Health England & Food Standards Agency , National Diet and Nutrition Survey. Rolling Programmes Years 9 to 11 (2016/20174 to 2018/2019) (Public Health England and Food Standards Agency, 2020).

[40] HM Government , “Childhood Obesity: Plan for Action,” 2016, https://www.gov.uk/government/publications/childhood-obesity-a-plan-for-action/childhood-obesity-a-plan-for-action.

[41] K. Hashem , H. Burt , M. Brown , and G. MacGregor , “Outcomes of Sugar Reduction Policies, United Kingdom of Great Britain and Northern Ireland,” Bulletin of the World Health Organization 102, no. 6 (2024): 432–439. 38812797 10.2471/BLT.23.291013 PMC11132159

[42] C. E. Evans and J. E. Cade , “A Cross‐sectional Assessment of Food‐ And Nutrient‐Based Standards Applied to British Schoolchildren's Packed Lunches,” Public Health Nutrition 20, no. 3 (2017): 565–570. 27609059 10.1017/S1368980016002251 PMC10261496

[43] C. J. Wall and J. Pearce , “Energy and Nutrient Content of School Lunches Provided for Children Attending School‐based Nurseries: A Cross‐sectional Study,” Public Health Nutrition 26, no. 12 (2023): 2641–2651. 37921199 10.1017/S1368980023002331 PMC10755416

[44] J. Pearce and C. J. Wall , “School Lunch Portion Sizes Provided for Children Attending Early Years Settings Within Primary Schools: A Cross‐Sectional Study,” Journal of Human Nutrition and Dietetics 36, no. 5 (2023): 1887–1900. 37278164 10.1111/jhn.13183

[45] R. E. Conway , G. N. Heuchan , L. Heggie , et al., “Ultra‐Processed Food Intake in Toddlerhood and Mid‐Childhood in the UK: Cross Sectional and Longitudinal Perspectives,” European Journal of Nutrition 63, no. 8 (2024): 3149–3160. 39363048 10.1007/s00394-024-03496-7 PMC11519182

[46] D. Neri , E. M. Steele , N. Khandpur , et al., “Ultraprocessed Food Consumption and Dietary Nutrient Profiles Associated With Obesity: A Multicountry Study of Children and Adolescents,” Obesity Reviews 23, no. 1 (2022): e13387. 34889015 10.1111/obr.13387

[47] J. C. Parnham , K. Chang , F. Rauber , et al., “The Ultra‐Processed Food Content of School Meals and Packed Lunches in the UK, 2008–17: A Pooled Cross‐Sectional Study,” The Lancet 400 (2022): S12. 10.1016/S0140-6736(22)02222-X 36929954

[48] B. O'Rourke , A. Shwed , B. Bruner , and K. Ferguson , “What's for Lunch? Investigating the Experiences, Perceptions, and Habits of Parents and School Lunches: A Scoping Review,” Journal of School Health 90, no. 10 (2020): 08–20. 10.1111/josh.12944 32820557

